# Survival outcomes and prognostic factors in *de novo* advanced biliary tract cancer: insights from a Saudi multicenter cohort

**DOI:** 10.3389/fonc.2026.1712037

**Published:** 2026-06-29

**Authors:** Bader Alshamsan, Emad Tashkandi, Mohammed Alghamdi, Ali H. Alfakeeh, Ali M. Alzahrani, Abdulhameed H. Alfagih, Mohammad Alkaiyat, Syed Zia Ul Hasan, Husam Shehata, Reham Alghandour, Mohammad Zuhdy, Ahmed Abdelhalim, Mohammed A. Algarni, Kanan Alshammari

**Affiliations:** 1 Department of Medicine, College of Medicine, Qassim University, Qassim, Saudi Arabia; 2 Prince Faisal Cancer Center, King Fahad Specialist Hospital, Qassim Health Cluster, Buraydah, Saudi Arabia; 3 College of Medicine, Umm Al-Qura University, Makkah, Saudi Arabia; 4 Oncology Center, King Saud University Medical City, King Saud University, Riyadh, Saudi Arabia; 5 Comprehensive Cancer Center, Medical Oncology, King Fahad Medical City, Riyadh, Saudi Arabia; 6 Department of Medical Oncology, King Abdulaziz Medical City, Ministry of National Guard for Health Affairs, Riyadh, Saudi Arabia; 7 Medical Oncology Department, King Abdullah Medical City, Makkah, Saudi Arabia; 8 Medical Oncology Department, Oncology Center, Mansoura University, Mansoura, Egypt; 9 Surgical Oncology Department, King Abdullah Medical City, Makkah, Saudi Arabia; 10 Surgical Oncology Department, Oncology Center, Mansoura University, Mansoura, Egypt; 11 Department of Medicine, King Saud bin Abdulaziz University for Health Sciences, Riyadh, Saudi Arabia; 12 King Abdullah International Medical Research Center, Riyadh, Saudi Arabia

**Keywords:** biliary tract cancer, cholangiocarcinoma, gallbladder cancer, molecular profiling, prognostic factors, Saudi Arabia, survival outcomes, systemic therapy

## Abstract

**Purpose:**

Advanced biliary tract cancer (BTC) carries a poor prognosis, with limited regional data available. This study evaluated outcomes, treatment patterns, and prognostic factors in a multicenter cohort of patients with *de novo* advanced BTC.

**Methods:**

A retrospective cohort of 271 patients with *de novo* advanced biliary tract cancer diagnosed at five tertiary centers in Saudi Arabia (2016–2023) was analyzed. Clinical, pathological, treatment, and molecular data were extracted from electronic health records. Overall survival was estimated using the Kaplan–Meier analysis, and prognostic factors were assessed through multivariable Cox regression.

**Results:**

Systemic therapy was administered to 211 patients (77.9%). Among the entire cohort, 92 patients (33.9%) received second-line therapy, 25 (9.2%) received third-line therapy, and 8 (3.0%) received fourth-line therapy. Median overall survival (OS) was 9.5 months (95% CI, 8.4–10.6). On multivariable analysis, inferior OS was independently associated with poor ECOG performance status (PS 4: HR 4.08, P = 0.031), age 15–39 years (HR 4.44, P < 0.001), extrahepatic cholangiocarcinoma (HR 1.91, P = 0.017), cirrhosis (HR 3.13, P = 0.004), and elevated CEA (HR 1.92, P = 0.003). Patients with disease control had a 71% lower hazard of death (HR 0.29, 95% CI 0.18–0.47) and longer median OS (16.0 vs 7.7 months).

**Conclusions:**

Advanced BTC in Saudi Arabia demonstrates poor outcomes, with survival strongly influenced by performance status, age, tumor site, cirrhosis, and biomarkers. Treatment exposure was limited, with only one-third receiving second-line therapy, highlighting the urgent need for expanded access to immunotherapy and molecular profiling.

## Introduction

Biliary tract cancers (BTC) are typically classified by their anatomical site, including intrahepatic cholangiocarcinoma (ICC) arising above the second-order bile ducts, extrahepatic cholangiocarcinoma (ECC) encompassing perihilar and distal forms, gallbladder adenocarcinoma (GBC), and other uncommon biliary tumors (1, 2). Although cholangiocarcinoma is a rare cancer, its global mortality has steadily increased over the past two decades. Annual incidence varies widely, from 0.3–6 per 100,000 in Western countries to >6 in parts of East Asia, with extremes of 85 per 100,000 in northeastern Thailand and 0.35 per 100,000 in Canada (3, 4). In Saudi Arabia (SA), the burden of BTC escalated markedly between 1990 and 2019, with incident cases increasing by more than 300%, deaths rising by 100–200%, and DALYs increasing by 200–300% (5). Obesity and gallstone disease (6), established risk factors for BTC, have increased in SA and may contribute to this trend. According to the most recent Saudi Cancer Registry report (2022), the age-standardized incidence for GBC was 1.2 per 100,000 in females and 1 per 100,000 in males (7).

Despite therapeutic advances, advanced biliary tract cancer remains a challenging disease, with a median overall survival (OS) typically less than one year with single-agent chemotherapy. Combination therapy with gemcitabine and cisplatin has modestly improved outcomes, extending survival to approximately 11.2 months vs. 7.7 months (8). In 2022 and 2023, phase III TOPAZ-1 and KEYNOTE-966 trials demonstrated that the addition of the immune checkpoint inhibitors durvalumab or pembrolizumab to gemcitabine-cisplatin further improved survival compared with chemotherapy alone, with median survival reaching 12–13 months, thereby establishing chemo-immunotherapy as a new standard of care in the first-line treatment of advanced BTC (9–11). Current clinical guidelines now endorse the use of cisplatin and gemcitabine in combination with either durvalumab or pembrolizumab as preferred first-line treatment options (12). Growing research into the molecular profile of BTC has identified several actionable genetic alterations, including FGFR2 fusions (13), IDH1/IDH2 mutations (14), ERBB2 amplifications (2), NTRK and RET gene fusions, KRAS and TP53 mutations (15), laying the foundation for personalized therapeutic approaches. Nevertheless, the integration of molecular testing and targeted therapies into routine care remains uneven globally, primarily due to disparities in access to diagnostic tools and treatment options.

Tumor location, patient sex, ECOG performance status, number of metastatic sites, and a range of biochemical markers, including bilirubin, white blood cell and neutrophil counts, hemoglobin, neutrophil-to-lymphocyte ratio (NLR), CA 19-9, and carcinoembryonic antigen (CEA), have all been identified as independent prognostic factors in BTC (16–20). However, treatment outcomes can vary widely depending on regional differences in epidemiology, tumor biology, and healthcare infrastructure. In the Middle East, and particularly in SA, there is a notable scarcity of published data on BTC. As a result, key aspects of the disease, such as clinical presentation, treatment patterns, and survival outcomes, remain poorly understood in this population. This lack of region-specific data hinders informed clinical decision-making and health policy development. To address this gap, we conducted a multicenter retrospective cohort study across five tertiary cancer centers in SA. Our objective was to characterize the clinical features of advanced BTC, evaluate real-world treatment patterns, and analyze survival outcomes and associated prognostic factors in this population.

## Patients and methods

This retrospective multicenter cohort study was conducted across five tertiary cancer centers in SA: the Ministry of National Guard Health Affairs, King Fahad Medical City, Prince Faisal Cancer Center, King Saud University Cancer Center, and King Abdullah Medical City Cancer Center. The study protocol was approved by the Institutional Review Boards (IRBs) of the Ministry of National Guard Health Affairs (IRB number 2397/23) on September 25, 2023, and conducted in accordance with the Declaration of Helsinki and local ethical regulations. Given the retrospective design and use of de-identified data, informed consent was waived by the IRB.

Consecutive patients diagnosed between 2016 and 2023 were screened for eligibility. Eligible patients were adults (≥18 years) with histologically confirmed BTC, including ICC, ECC, and GBC. Only patients with unresectable or *de novo* metastatic disease at the time of initial diagnosis were included. *De novo* metastatic disease was defined as the presence of distant metastases at the time of initial histologic diagnosis, without prior curative-intent surgery or adjuvant therapy. Patients who developed metastases after resection or adjuvant treatment were excluded, as were those with incomplete clinical data. As this was an exploratory retrospective study, no formal sample size calculation was performed. Instead, all consecutive patients meeting these eligibility criteria during the study period were included, ensuring a comprehensive and representative sample of real-world patients.

Clinical and pathological data were abstracted from electronic health records using a standardized case report form, following the STROBE reporting guidelines for observational studies (21). Variables collected included demographic information (age at diagnosis, sex, nationality, body mass index [BMI, categorized as underweight, normal weight, overweight, or obese], smoking status, and alcohol use), baseline Eastern Cooperative Oncology Group (ECOG) performance status, and comorbidities including hypertension, diabetes mellitus (DM), cardiac disease, cirrhosis, chronic kidney disease, and others. Tumor characteristics encompassed primary site (ICC, ECC, or GBC), grade, lymphovascular and perineural invasion, and biomarker expression. Laboratory parameters included tumor markers, CA 19-9 (<37 U/mL vs >37 U/mL), CEA (≤5 ng/mL vs > 5 ng/mL), and alpha-fetoprotein (AFP) (< 10 ng/mL vs. ≥ 10 ng/mL) (20), and systemic inflammatory markers, NLR (≥3 vs <3), based on prior BTC literature (16). Where available, molecular profiling was retrieved from next-generation sequencing (NGS), capturing alterations in FGFR2, IDH1, KRAS/NRAS, HER2, BRAF, and other recurrent or actionable genes.

Treatment-related information included the type of systemic therapy (chemotherapy, targeted therapy, immunotherapy, or combinations), duration, response assessment, and reasons for discontinuation. Discontinuation was classified as progression, toxicity, planned completion, or others. Others comprised non-compliance, treating physician discretion, voluntary withdrawal, loss to follow-up, or unknown reasons. The use of radiotherapy was also recorded.

Study outcomes included OS, defined from the date of diagnosis of *de novo* metastatic disease to death from any cause or last follow-up. Median follow-up was estimated using the reverse Kaplan–Meier method. Progression-free survival (PFS) was defined as the time from treatment initiation to radiographic or clinical progression (as per RECIST v1.1 or the treating physician) or death. Objective response rate (ORR) was defined as the proportion of patients achieving complete or partial response, while disease control rate (DCR) included complete response, partial response, or stable disease as best response according to RECIST v1.1 criteria. Response assessments were investigator-assessed in routine practice and were not centrally reviewed. Patients alive or progression-free at the time of analysis were censored at their last known follow-up date, with survival updated through July 2025.

### Statistical analysis

Statistical analyses were performed using SPSS for Mac, Version 30 (IBM Corp., Armonk, NY, USA). Continuous variables were summarized as medians with interquartile ranges (IQRs), and categorical variables as counts and percentages. Data cutoff: July 31, 2025.

Survival distributions were estimated using the Kaplan–Meier method and compared using the log-rank test. For exploratory analyses, OS was stratified by the actual number of systemic therapy lines delivered (best supportive care only; exactly 1; exactly 2; exactly 3; and exactly 4 lines), based on complete treatment records. Treatment-line exposure was analyzed as a stratification variable and was not modeled as a time-dependent covariate. Variables with a p-value <0.10 in univariable analyses were included in multivariable Cox proportional hazards regression. Hazard ratios (HRs) with 95% confidence intervals (CIs) were reported. A two-sided p-value <0.05 was considered statistically significant. Biomarker analyses were performed among patients with available data for each marker. Missing data were minimal and excluded from analyses by listwise deletion, and no imputation was performed.

## Results

### Patients and disease characteristics

A total of 271 patients with *de novo* metastatic biliary tract cancer were included. The median age was 62 years (IQR, 54–70), with 15 patients (5.5%) classified as AYAs (15–39 years) and 108 (39.9%) as elderly (≥65 years). Women accounted for 169 patients (62.3%), and 249 (91.9%) were Saudi nationals. The most common primary tumor sites were ICC (114, 42.1%) and GBC (114, 42.1%), followed by ECC (39, 14.4%). Most tumors were moderately or poorly differentiated (grade 2: 106, 39.5%; grade 3: 138, 50.9%). At baseline, 128 patients (47.4%) had ECOG performance status (PS) 0–1, while 73 (27.0%) had PS 3–4. Comorbidities were frequent, with hypertension (137, 50.9%) and DM (128, 47.2%) most common, followed by cardiac disease (36, 13.3%), cirrhosis (18, 6.6%), and chronic kidney disease (17, 5.9%). Detailed baseline characteristics are summarized in **Table 1**.

**Table 1:** Characteristics of the patients with *de novo* metastatic biliary tract cancer (n=271).

Characteristic		No. (%)
Age, Median, (IQR)		62 (54–70)
	AYA (15-39)	15 (5.5)
	Older Adults (40-64)	145 (53.9)
	Elderly ≥65	108 (39.9)
	NA	3 (1.1)
Sex	Male	102 (37.6)
	Female	169 (62.3)
Nationality	Saudi	249 (91.9)
	Non-Saudi	22 (8.1)
Primary tumor site	Intrahepatic cholangiocarcinoma	114 (42.1)
	Gallbladder adenocarcinoma	114 (42.1)
	Extrahepatic cholangiocarcinoma	39 (14.4)
	Other biliary tumors	4 (1.5)
Tumor grade	Grade 1	11 (4.1)
	Grade 2	106 (39.5)
	Grade 3	138 (50.9)
	NA	16 (5.9)
ECOG PS	0/1	128 (47.4)
	2	69 (25.5)
	3	42 (15.5)
	4	31 (11.4)
	NA	1 (0.4)
Comorbidities	Diabetes mellitus	128 (47.2)
	Hypertension	137 (50.9)
	Cardiac disease	36 (13.3)
	Chronic kidney disease	17 (5.9)
	Cirrhosis	18 (6.6)
	Others*	72 (26.6)
BMI category	Underweight	16 (5.9)
	Normal weight	107 (39.5)
	Overweight	69 (25.5)
	Obese	76 (28.4)
	NA	3 (1.1)

*Other comorbidities included history of stroke (n = 8, 3.0%), chronic viral hepatitis [HBV, n = 4 (1.5%); HCV, n = 5 (1.8%)], thromboembolic disease [PE/DVT/portal/renal/iliac vein thrombosis, n = 7 (2.6%)], chronic pulmonary disease [asthma/COPD, n = 5 (1.8%)], and autoimmune/inflammatory conditions [ulcerative colitis, n = 3 (1.1%); inflammatory bowel disease, n = 2 (0.7%)]. A range of additional conditions were each reported in a single patient (<1%), including bipolar disorder, epilepsy, rheumatoid arthritis, and hypothyroidism. AYA, adolescent and young adult; BMI, body mass index; ECOG PS, Eastern Cooperative Oncology Group performance status; IQR, interquartile range; NA, not available.

Among evaluable patients, CA 19–9 was elevated in 168 of 237 (70.9%), CEA in 98 of 211 (46.4%), and AFP in 35 of 158 (22.2%). A high neutrophil-to-lymphocyte ratio (≥3) was observed in 146 of 265 (55.1%). NGS was performed in 32 patients (11.9%), of whom 17 (53.1%) harbored actionable alterations. In ICC, the most frequent findings were FGFR2 fusions/rearrangements (n = 4, 12.5%) and IDH1 mutations (n = 2, 6.3%), followed by KRAS (n = 2, 6.3%) and NRAS (n = 2, 6.3%). In GBC, HER2 amplification was detected in 3 patients and CCND1 amplification in 1 patient; an additional HER2 amplification was seen in one ICC case. Rare events (<5% each) included alterations in PIK3CA, MYC, SMAD4, STK11, TP53, and RICTOR. No actionable alterations were identified in ECC (n = 2).

### Systemic therapy

Of the 271 patients, 211 (77.9%) received first-line systemic therapy, and 60 (22.1%) did not, largely due to poor performance status or patient preference. Subsequent therapy was initiated in 92/271 (33.9%) for second line, 25/271 (9.2%) for third, and 8/271 (3.0%) for fourth. First-line therapy was dominated by gemcitabine–platinum regimens, particularly gemcitabine–cisplatin with or without durvalumab. Gemcitabine monotherapy and fluoropyrimidine-based regimens were also used. In the second line, fluoropyrimidine-based therapies predominated (70%), with fewer patients receiving gemcitabine rechallenge, targeted therapies, or immunotherapy. In the third line, most patients received fluoropyrimidines, while only a minority received targeted therapy (T-DXd, pemigatinib, erdafitinib, T-DM1) or immunotherapy. In the fourth line, treatment was fluoropyrimidine, gemcitabine, or HER2-directed therapy.

### Treatment efficacy and survival outcomes

Among patients receiving systemic therapy, the overall response rate (ORR) was 19.4% and the disease control rate (DCR) was 40.8% (**Table 2**). First-line regimens demonstrated variable efficacy: ORR ranged from 13.0% with gemcitabine monotherapy to 47.6% with gemcitabine–cisplatin plus durvalumab, with corresponding DCRs of 23.9% and 66.7%, respectively. FOLFIRINOX achieved an ORR of 40.0% and DCR of 60.0%, though only five patients received this regimen. In the second line, pooled outcomes declined (ORR 16.3%, DCR 38.0%), but FOLFOX showed activity (ORR 23.8%, DCR 66.7%), and targeted therapies in molecularly selected patients achieved a DCR of 50.0%. By the third and fourth lines, benefit was modest overall, but durable disease control was observed in select patients receiving targeted or HER2-directed therapies. Treatment discontinuation was most often due to progression, followed by toxicity, with fewer cases of planned completion or other causes (non-compliance, withdrawal, or at the treating physician’s discretion). The detailed type of therapy, efficacy, and reasons for discontinuation are presented in **Table 2**.

**Table 2:** Systemic therapy used in BTC patients according to the treatment line.

Line of therapy	Regimen	n (%)	Median duration (mo) [IQR]	ORR* (%)	DCR* (%)	Reason for discontinuation
First-line (n=211)	All first line regimens pooled	211/271 (77.9%)	2.9 [1.8–4.9]	19.4	40.8	PD: 122, Toxicity: 48, Completion: 19, Others**: 22
	Gemcitabine-cisplatin	114 (54.0)	3.3 [1.9–4.9]	17.5	42.1	PD 63, Toxicity 26, Completion 13, Others 12
	Gemcitabine-cisplatin plus durvalumab	21 (10.0)	6.4 [2.9–11.6]	47.6	66.7	PD 16, Toxicity 3, Completion 1, Others 1
	Gemcitabine-carboplatin	20 (9.5)	2.7 [1.3–4.5]	10.0	30.0	PD 12, Toxicity 5, Completion 2, Others 1
	FOLFIRINOX	5 (2.4)	6.0 [1.6–14.2]	40.0	60.0	PD 3, Completion 1, Others 1
	Gemcitabine monotherapy	46 (21.8)	2.1 [1.4–3.6]	13.0	23.9	PD 25, Toxicity 12, Completion 2, Others 7
	Others	5 (2.4)	2.9 [2.8–8.1]	20.0	80.0	PD 3, Toxicity 2
Second-line (n=92)	All second line regimens pooled	92/271 (33.9%)	2.1 [1.4–3.5]	16.3	38.0	PD 53, Toxicity 27, Completion 1, Others 11
	FOLFOX	21 (22.8)	2.4 [1.4–3.6]	23.8	66.7	PD 12, Toxicity 5, Completion 1, Others 3
	Other fluoropyrimidine (Capecitabine, CAPIRI, etc.)	44 (47.8)	2.1 [1.3–3.5]	15.9	27.3	PD 25, Toxicity 13, Others 5
	Gemcitabine-based	11 (12.0)	1.9 [1.6–4.7]	18.2	36.4	PD 9, Toxicity 1, Others 1
	Fluoropyrimidine plus gemcitabine	8 (8.7)	2.0 [1.4–3.0]	0.0	42.9	PD 3, Toxicity 3, Others 2
	Immunotherapy	2 (2.2)	1.0 [0.9–1.0]	0.0	0.0	Toxicity 2
	Targeted therapy (FGFR2, IDH1, HER2, T-DXd)	4 (4.4)	3.7 [1.7–5.6]	25.0	50.0	PD 4
	Others	2 (2.2)	1.2 [1.2–1.2]	0.0	0.0	Toxicity 2
Third line (n=25)	All third line regimens pooled	25/271 (9.2%)	2.3 [1.3–3.3]	16.0	32.0	PD: 18, Toxicity: 3, Others: 4
	Targeted therapy (T-DXd, erdafitinib, pemigatinib, T-DM1)	4 (16.0)	3.8 [3.0–10.2]	25.0	50.0	PD 4
	Immunotherapy	2 (8.0)	2.0 [1.9–2.1]	0.0	50.0	PD 1, Toxicity 1
	Fluoropyrimidine-based	19 (76.0)	2.1 [1.2–3.2]	15.8	26.3	PD 13, Toxicity 2, Others 3
Fourth line (n=8)	All fourth line regimens pooled	8/271 (3.0%)	2.9 [1.7–3.5]	25.0	62.5	PD: 5, Toxicity: 2, Others: 1
	Targeted therapy (T-DXd, capecitabine + trastuzumab)	2 (25.0)	2.2 [1.4–3.0]	50.0	50.0	PD 1, Others 1
	Fluoropyrimidine-based	2 (25.0)	3.1 [2.5–3.6]	0.0	100.0	PD 2
	Gemcitabine-based	4 (50.0)	2.9 [1.6–4.5]	25.0	50.0	PD 2, Toxicity 2

*ORR (objective response rate) and DCR (disease control rate) were calculated based on the best radiographic response per RECIST v1.1. In contrast, discontinuation reasons reflect the eventual cause of treatment cessation for the entire cohort. Patients achieving disease control may remain on therapy for a period (median duration shown) but will ultimately discontinue, most often due to progression. Therefore, ORR/DCR values are not expected to match the distribution of discontinuation reasons. **Others comprised non-compliance, investigator discretion, voluntary withdrawal, lost to follow-up, or unknown reasons.

BTC, biliary tract cancer; CAPIRI, capecitabine + irinotecan; DCR, disease control rate; FOLFIRINOX, fluorouracil + leucovorin + irinotecan + oxaliplatin; FOLFOX, fluorouracil + leucovorin + oxaliplatin; GemCis, gemcitabine + cisplatin; IQR, interquartile range; mo, months; ORR, objective response rate; PD, progressive disease; T-DM1, trastuzumab emtansine; T-DXd, trastuzumab deruxtecan.

**Figure 1:**
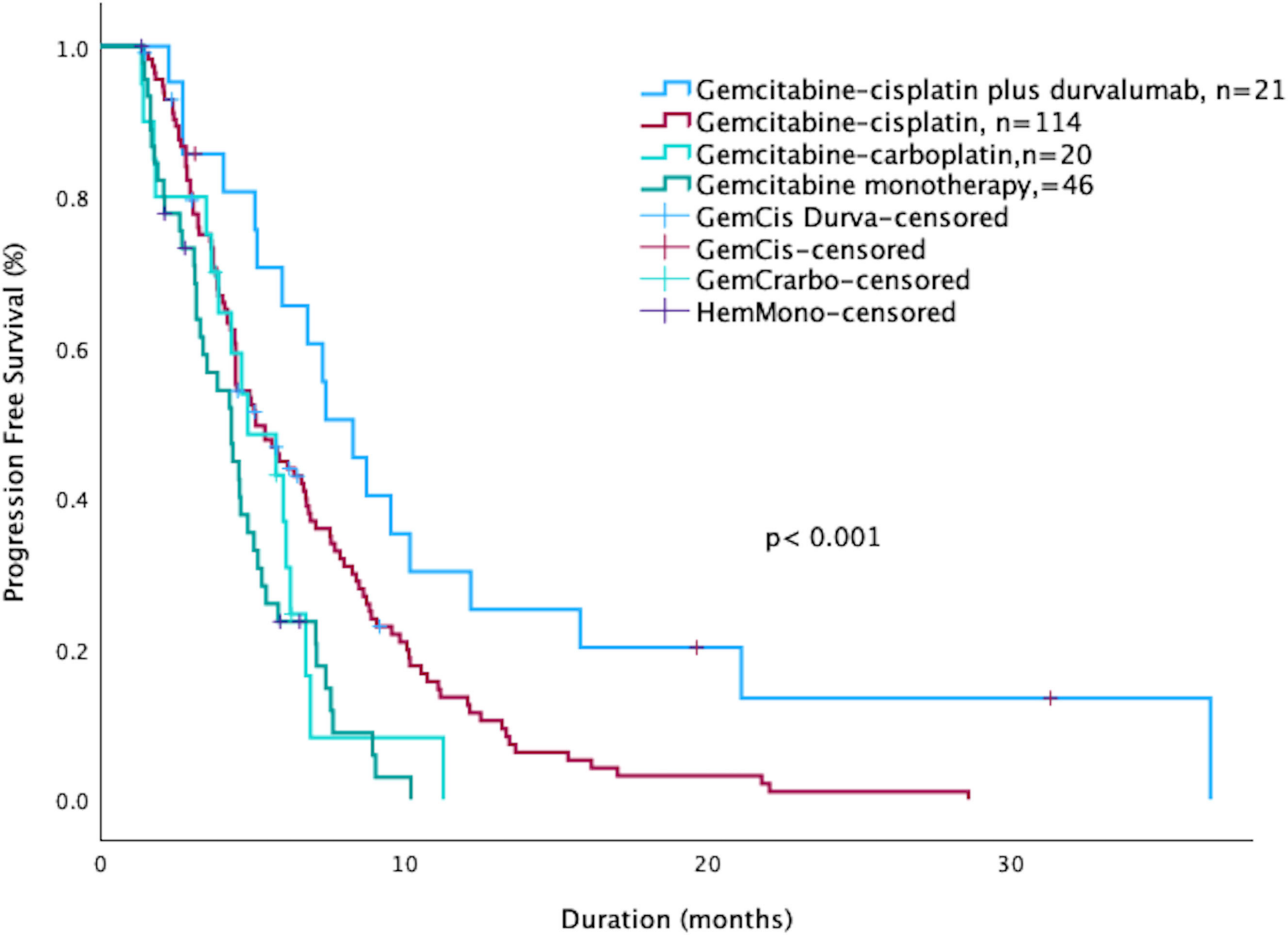
Kaplan–Meier curves of progression-free survival (PFS) by first-line regimen.

With a median follow-up of 19.6 months (95% CI, 16.4–22.8), the median overall survival (OS) for the whole cohort was 9.5 months (95% CI, 8.4–10.6). Median PFS differed significantly across first-line regimens (log-rank P < 0.001; **Figure 1**). Median PFS was 5.1 months (95% CI, 3.8–6.4) for gemcitabine–cisplatin, 8.3 months (95% CI, 6.3–10.4) for gemcitabine–cisplatin plus durvalumab, 4.9 months (95% CI, 2.8–6.9) for gemcitabine–carboplatin, and 4.3 months (95% CI, 3.3–5.4) for gemcitabine monotherapy. Small subgroups treated with FOLFIRINOX (n=5) or other regimens (n=5) were excluded from PFS analysis due to limited sample size.

**Figure 2:**
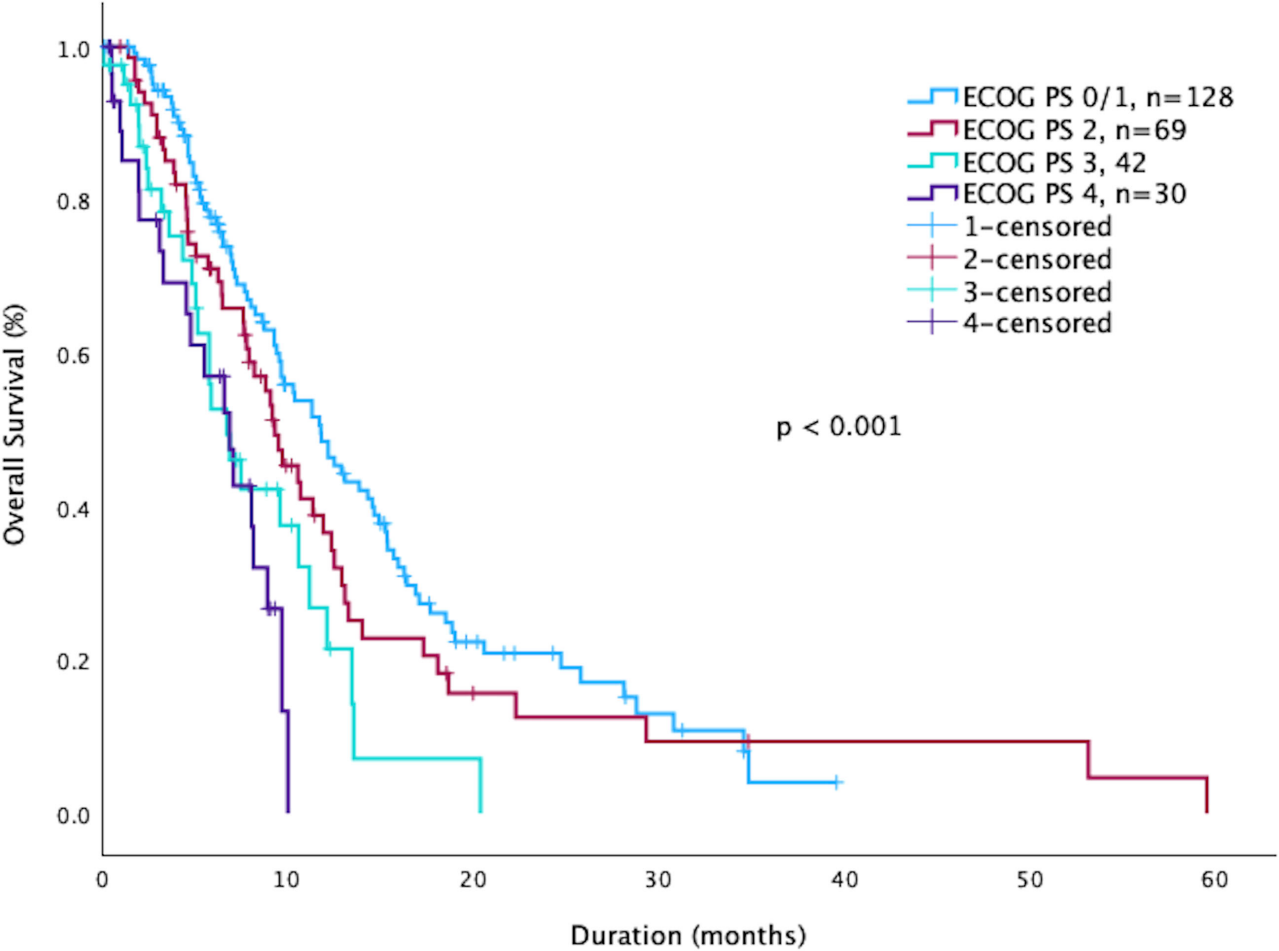
Kaplan–Meier curves of overall survival (OS) stratified by baseline ECOG performance status.

On Kaplan–Meier analysis, median OS did not differ significantly by age group (AYA, 9.3 months; older adults, 9.3 months; elderly, 10.0 months; P = 0.66), sex (male, 9.7 months; female, 9.5 months; P = 0.54), BMI category (P = 0.24), or tumor markers (CA 19-9, AFP, CEA). However, in multivariable Cox regression, AYA status and elevated CEA levels (>5 ng/mL) were independently associated with inferior survival (HR 4.44, p < 0.001 and HR 1.92, p = 0.003, respectively). ECOG performance status was strongly prognostic (P < 0.001; **Figure 2**): patients with PS 0–1 had a median OS of 11.8 months, compared with 9.3 months for PS 2, 6.7 months for PS 3, and 6.9 months for PS 4. In multivariable Cox regression, ECOG PS did not retain global statistical significance, but PS 4 remained an independent adverse factor (HR 4.08, p = 0.031).

**Figure 3:**
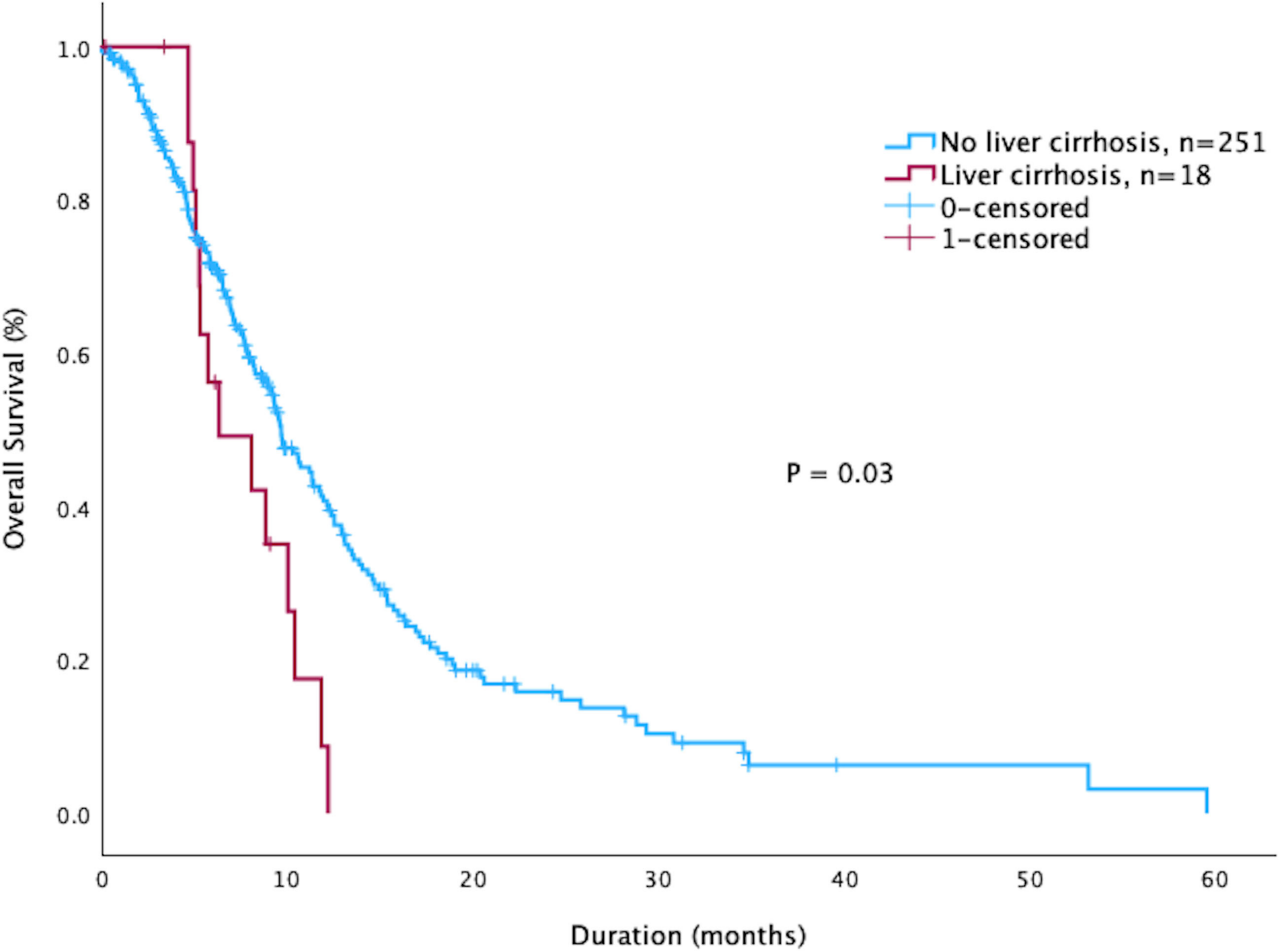
Kaplan–Meier curves of OS stratified by the presence of cirrhosis.

A history of cirrhosis was also associated with inferior survival (6.3 vs. 9.7 months; P = 0.03; **Figure 3**). Elevated NLR (≥3) showed a trend toward worse outcomes (median OS 8.2 vs. 11.4 months; P = 0.06). Median OS varied across first-line regimens, though differences did not reach statistical significance (log-rank P = 0.10). Outcomes were 10.3 months (95% CI, 8.2–12.4) with gemcitabine–cisplatin, 11.8 months (95% CI, 4.9–18.7) with gemcitabine–cisplatin plus durvalumab, 10.4 months (95% CI, 8.5–12.4) with gemcitabine–carboplatin, and 9.8 months (95% CI, 6.0–13.6) with gemcitabine monotherapy. Patients treated with FOLFIRINOX (n=5) achieved a median OS of 24.7 months, while those on other regimens had 8.0 months; however, these subgroups were too small for statistical comparison.

**Figure 4:**
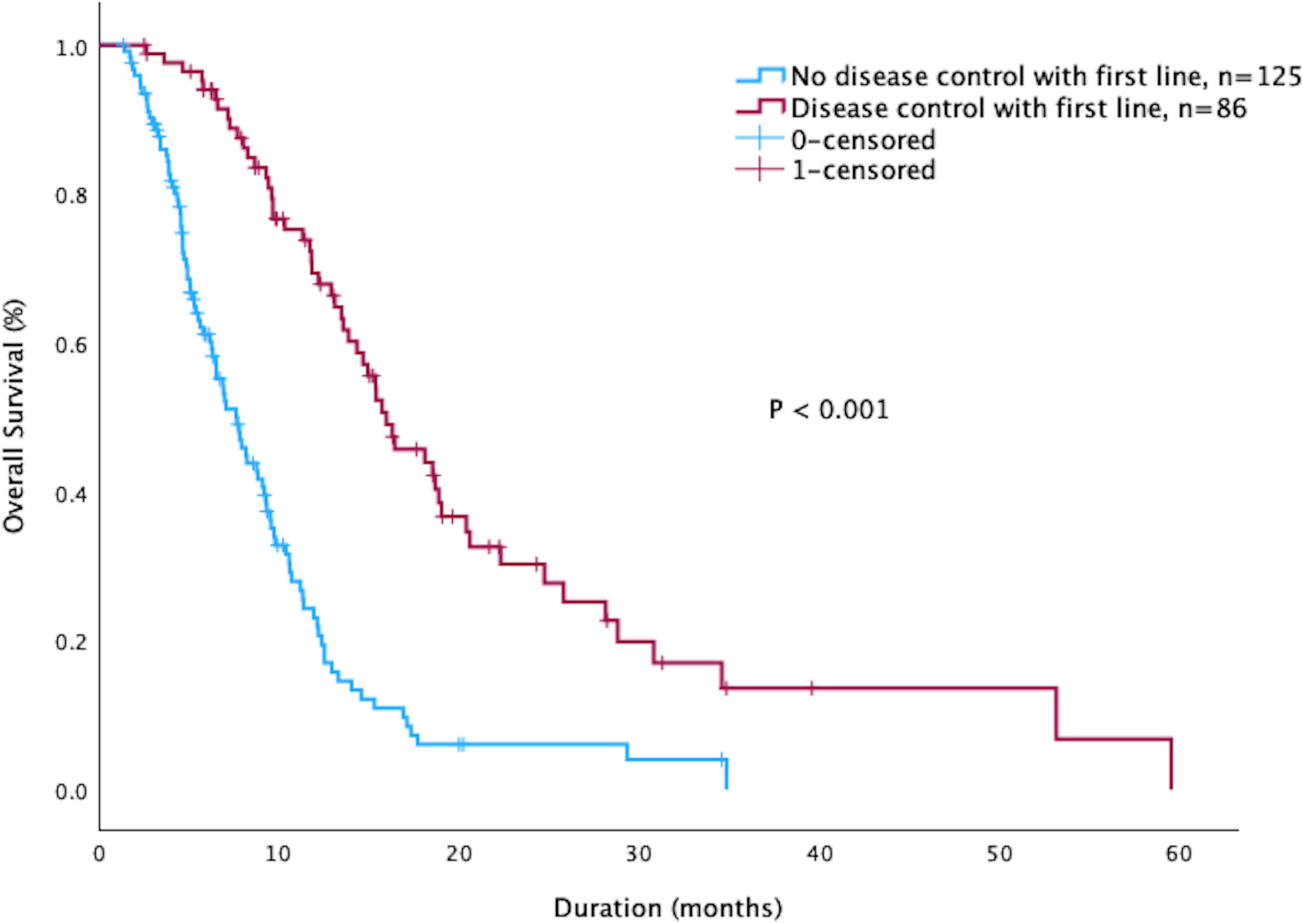
Kaplan–Meier curves of OS according to disease control to first-line treatment response.

**Figure 5:**
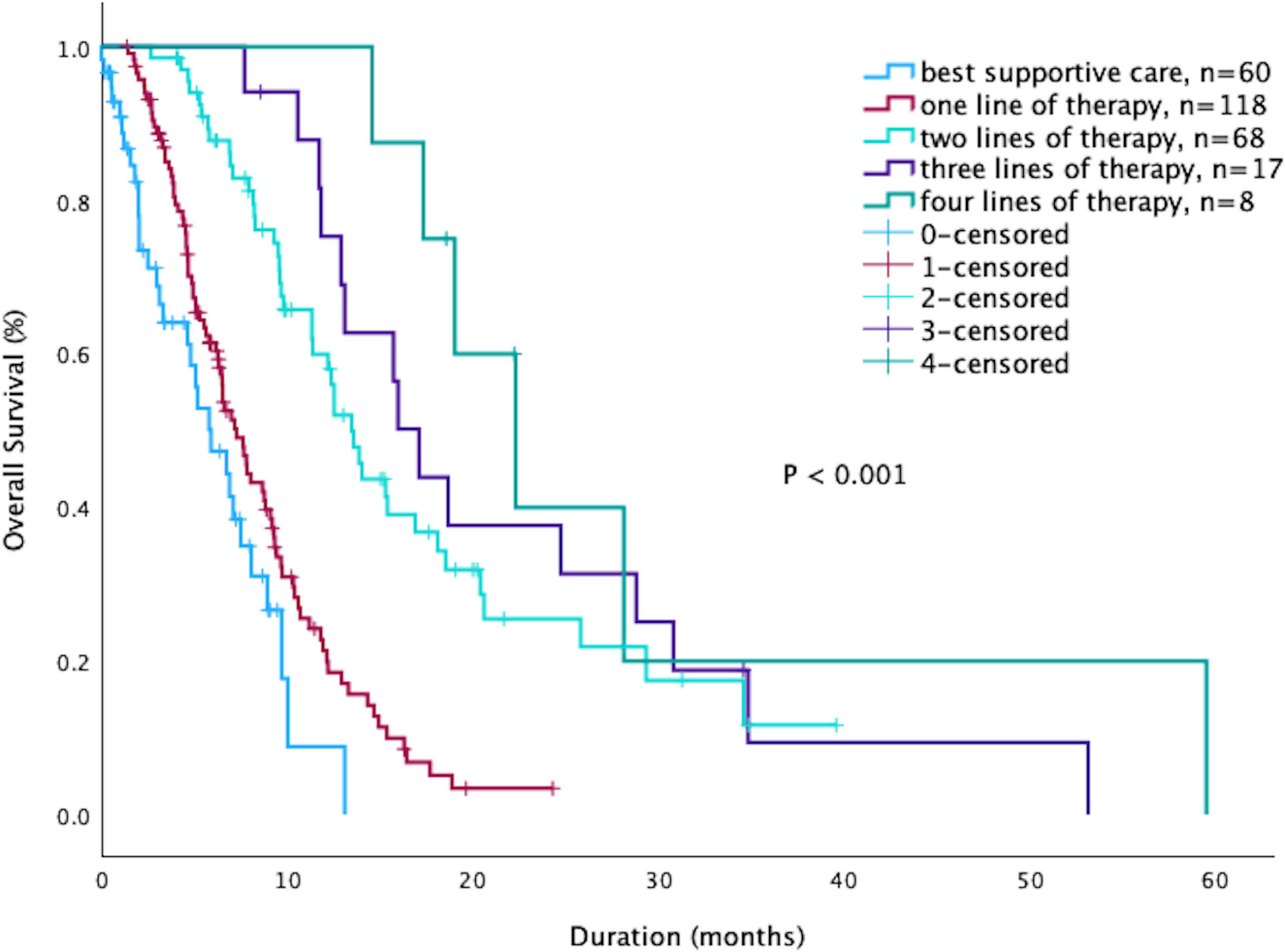
Kaplan–Meier curves of OS by number of therapy lines.

Treatment response to first-line therapy was highly predictive: patients achieving disease control had a median OS of 16.0 months versus 7.7 months without disease control (P < 0.001; **Figure 4**). In exploratory descriptive analyses, OS increased stepwise according to the number of therapy lines (P < 0.001; **Figure 5**): best supportive care (5.9 months), one line (7.3 months), two lines (13.5 months), three lines (17.1 months), and four lines (22.3 months). Detailed estimates of OS for all variables are provided in **Table 3**.

**Table 3:** Univariate and multivariate Cox regression analysis of overall survival (n = 271).

Variable	HR (95% CI)	P value	HR (95% CI)	P value
Age (years)		0.665 (overall)		<0.001 (overall)
40–64 (ref)	1.00	–	1.00	–
15–39	1.14 (0.61–2.12)	0.688	4.44 (1.84–10.73)	<0.001
≥65	0.89 (0.65–1.22)	0.471	0.81 (0.50–1.31)	0.389
Sex				
Male (ref)	1.00	–	1.00	–
Female	0.91 (0.53–1.57)	0.728	0.96 (0.65–1.42)	0.836
Primary tumor site		0.082 (overall)		0.081 (overall)
Intrahepatic (ref)	1.00	–	1.00	–
Extrahepatic	1.22 (0.80–1.85)	0.354	1.91 (1.12–3.24)	0.017
Gallbladder	1.55 (1.11–2.15)	0.010	1.45 (0.91–2.32)	0.118
Other	1.20 (0.29–4.93)	0.801	Not estimable*	–
ECOG PS		<0.001 (overall)		0.171 (overall)
0–1 (ref)	1.00	–	1.00	–
2	1.26 (0.89–1.80)	0.199	0.99 (0.61–1.62)	0.980
3	2.10 (1.34–3.30)	0.001	1.32 (0.61–2.85)	0.476
4	2.85 (1.69–4.80)	<0.001	4.08 (1.14–14.60)	0.031
Cirrhosis				
No (ref)	1.00	–	1.00	–
Yes	1.83 (1.05–3.18)	0.034	3.13 (1.44–6.80)	0.004
CEA				
≤5 ng/mL (ref)	1.00	–	1.00	–
>5 ng/mL	1.37 (0.98–1.93)	0.069	1.92 (1.24–2.96)	0.003
NLR ≥3	1.33 (0.99–1.79)	0.056	0.93 (0.61–1.42)	0.740
DCR (1st line)				
No (ref)	1.00	–	1.00	–
Yes	0.31 (0.22–0.44)	<0.001	0.29 (0.18–0.47)	<0.001
Number of therapy lines		<0.001 (overall)		<0.001 (overall)
BSC (ref)	1.00	–	1.00	–
First line	0.58 (0.38–0.88)	0.010	0.37 (0.23–0.58)	<0.001
Second line	0.19 (0.11–0.31)	<0.001	0.27 (0.14–0.55)	<0.001
Third line	0.15 (0.07–0.28)	<0.001	0.10 (0.03–0.36)	<0.001
Fourth line	0.08 (0.03–0.22)	<0.001	Not estimable*	–

*Excluded from multivariable analysis due to low sample size.

BMI, body mass index; BSC, Best supportive care; CEA, carcinoembryonic antigen; CI, confidence interval; DCR, disease control rate; ECOG PS, Eastern Cooperative Oncology Group performance status; HR, hazard ratio; NLR, neutrophil-to-lymphocyte ratio; OS, overall survival; ref, reference.

## Discussion

In this multicenter cohort of 271 patients with *de novo* advanced BTC treated across five tertiary cancer centers in SA, median OS was 9.5 months, broadly consistent with historical outcomes reported for advanced BTC in real-world settings (22–24). Thus, the primary contribution of this study is to provide regional multicenter real-world data on epidemiology, treatment patterns, survival outcomes, and access gaps in advanced BTC in SA. Several findings merit emphasis. Prognosis was influenced by clinical status and biology, with adverse factors identified in multivariable models including AYA age group, ECC, cirrhosis, and elevated baseline CEA levels. Achieving disease control with first-line therapy and remaining eligible for subsequent treatment lines correlated with more prolonged survival.

Gemcitabine–platinum regimens dominated first-line treatment; however, only one in ten patients received gemcitabine–cisplatin plus durvalumab, and this subgroup had numerically longer PFS (8.3 months) and OS (11.8 months). These findings should be considered descriptive because of the small sample size and potential temporal and selection biases; however, they are directionally consistent with global phase III data (TOPAZ-1, KEYNOTE-966) (10, 11). The relatively low uptake in our cohort likely reflects calendar timing, temporal adoption lag, and barriers related to drug availability and reimbursement.

The cohort reflected known sex differences in BTC, with females comprising 62.3% of cases (25, 26). Age distribution also varied by subtype: among AYA patients (15–39 years), ICC predominated (80%) with no ECC observed; in elderly patients (≥65 years), GBC was most frequent (48.1%); and in older adults aged 40–64 years, there was a balanced distribution across subtypes. These trends align with global epidemiology, where BTC incidence increases with age and GBC disproportionately affects older individuals (27). By sex, subtype distribution mirrored international patterns: GBC was more common in females than males (45.5% vs. 34.7%), ECC was more frequent in males (19.8% vs. 11.4%), and ICC occurred at similar rates in both sexes (42.6% vs. 42.5%). These findings are consistent with global data showing that GBC disproportionately affects women, whereas ICC and ECC are more prevalent in men (28, 29).

By KM analysis, median OS was similar across age groups. However, in the multivariable model, AYA status was an independent adverse prognostic factor. This suggests that unadjusted survival curves may not have fully captured the adjusted association, possibly due to baseline confounding. This finding should be interpreted cautiously because the AYA subgroup was small, the confidence intervals were wide, and residual confounding or model instability cannot be excluded. In contrast, elderly patients did not demonstrate inferior outcomes in either unadjusted or adjusted analyses. Although counterintuitive, poorer outcomes in AYAs with BTC have been hypothesized to reflect distinct tumor biology, delayed diagnosis due to low suspicion, and/or treatment disparities (30, 31). This observation should be considered hypothesis-generating and warrants validation in larger cohorts; if confirmed, it would support deeper molecular and biological characterization of AYA BTC in the region.

The tumor site was a significant determinant of survival. The majority of prior studies have reported ICC as the subtype with the poorest outcomes (25, 32), while others identified ECC as associated with the worst survival outcomes (26). In this cohort, univariate analysis suggested worse outcomes for GBC and ECC compared with ICC; however, after adjustment, only ECC retained independent significance. These differences likely reflect geographic variation, patient case mix, and disparities in treatment access across populations (28). Baseline cirrhosis was independently associated with poorer overall survival in our cohort, in alignment with previous studies that have demonstrated its adverse prognostic impact in BTC (33–35). Biomarkers also carried prognostic value. Elevated CEA (>5 ng/mL) independently predicted shorter OS (HR 1.92), whereas CA 19–9 did not (36, 37). Elevated NLR (≥ 3) demonstrated a trend toward worse overall survival (median OS 8.2 vs. 11.4 months; P = 0.06), consistent with prior reports in the literature (16). By contrast, sex, BMI and common comorbidities such as DM and hypertension were not prognostic. Notably, DM did not impact OS in this cohort, diverging from findings in a large Korean database, emphasizing the importance of regionally contextualized prognostic models (38).

Although ECOG performance status did not retain global significance in the multivariable model, PS 4 remained an independent predictor of poor survival. Treatment-related factors were also prognostic. Achieving disease control after first-line therapy was a strong predictor of improved OS. Furthermore, OS increased stepwise with additional lines of systemic therapy, ranging from a median of 5.9 months with best supportive care only to 22.3 months with four lines. However, this association should be interpreted with caution because it is likely influenced by survivor bias and immortal time bias, as patients who live longer are inherently more likely to receive additional lines of therapy. Practice patterns also vary internationally: in our cohort, only one-third of patients (33.9%) received second-line therapy, compared with 15% in the ABC-02 trial and up to 75% in Japanese cohorts (18, 39). Beyond first-line, fluoropyrimidine-based regimens predominated, with FOLFOX demonstrating the highest activity among standard options (40). Targeted therapies (e.g., FGFR and HER2-directed agents) and immunotherapy were used in small, molecularly selected subsets, yielding encouraging disease control in later lines. These results, though limited by small numbers, are consistent with trial data and reinforce the clinical value of integrating routine molecular testing (41).

Despite NGS being performed in only 11.9% of patients, more than half harbored actionable alterations (FGFR2 fusions, IDH1 mutations, ERBB2 amplification, among others). However, because molecular profiling was available for only a small subset of patients, these findings should not be interpreted as representative of the broader Saudi BTC population. Rather, the main observation supported by our data is the limited uptake of molecular profiling in routine practice, highlighting a potential access gap. Prior studies have shown that molecular alterations in BTC may carry prognostic and therapeutic relevance, including adverse associations with KRAS, TP53, and SMAD4 mutations and more favorable outcomes with FGFR2, IDH1/2, and BAP1 alterations. Emerging evidence also suggests that KRAS/TP53 co-mutations may confer sensitivity to immunotherapy, while KRAS mutations alone are linked to resistance (15, 42). These literature-based findings provide context for the potential value of molecular testing. The low percentage of cases that underwent NGS could be explained by limited tissue availability at diagnosis, as many BTC cases are diagnosed using fine-needle aspiration or brushing/washing during ERCP. Cost, reimbursement, and lack of in-house NGS capabilities could also play a role. Implementing reflex molecular testing pathways and considering liquid biopsy when tissue is limited may represent practical strategies to improve access to biomarker-driven care in the region. A practical regional pathway could prioritize FGFR2/IDH1 testing in ICC, reflex HER2 assessment in GBC and select ICC/ECC, and use liquid biopsy when tissue is limited (43).

Strengths of this study include the multicenter design across major oncology hubs, a relatively large sample size for a rare cancer, and a focus on *de novo* disease, which improves cohort homogeneity. Limitations include the retrospective nature, potential residual confounding, and limited adoption of durvalumab-based regimens due to calendar timing. Moreover, some multivariable estimates, particularly those involving small subgroups such as AYA patients and ECOG PS 4, may be unstable and should be interpreted cautiously. The association between receipt of additional lines of therapy and longer survival is likely influenced by survivor bias and immortal time bias. Therefore, this finding should be interpreted as exploratory and descriptive rather than causal. Furthermore, the small size of some treatment subgroups (such as FOLFIRINOX and targeted therapy cohorts) limits definitive statistical comparison and generalizability.

Future efforts should prioritize prospective regional registries with standardized data elements, including time-dependent treatment exposure, reasons for non-treatment, and quality-of-life metrics. Embedding routine reflex molecular testing with predefined panels and tracking actionability will help define the real-world impact of precision oncology. The observed adverse survival signal in AYA patients warrants validation and correlative studies exploring genomic, transcriptomic, and environmental factors in this subgroup. Notably, the PFS observed with gemcitabine-cisplatin plus durvalumab in our cohort was descriptive and directionally consistent with global phase III data, supporting further evaluation of chemo-immunotherapy where access permits. To translate these findings into better outcomes, efforts should focus on expanding access to chemo-immunotherapy and molecularly targeted agents, as these represent important opportunities to address a regional policy and access gap in advanced BTC.

## Conclusions

Advanced BTC in SA continues to show poor survival, with prognosis shaped by PS, tumor site, cirrhosis, and select biomarkers. Longer survival was observed among patients who achieved early disease control and maintained eligibility for subsequent therapy, yet access to modern regimens remains limited. Broader implementation of chemo-immunotherapy and reflex NGS could improve access to modern treatment strategies, while the adverse survival signal in AYA patients requires validation in larger cohorts. Prospective multicenter registries will be critical to advance precision oncology and harmonize care across the region.

## Data Availability

The raw data supporting the conclusions of this article will be made available by the authors, without undue reservation.
